# Lessons learnt in the first year of an Australian pediatric cardio oncology clinic

**DOI:** 10.1186/s40959-023-00194-x

**Published:** 2023-12-07

**Authors:** Claudia Toro, Ben Felmingham, Mangesh Jhadav, David S. Celermajer, Andre La Gerche, John O’Sullivan, Sanjeev Kumar, Marion K. Mateos, Joy Fulbright, Dinisha Govender, Lane Collier, Michael Cheung, David D. Eisenstat, Peter W. Lange, Julian Ayer, David A. Elliott, Rachel Conyers

**Affiliations:** 1 Cardiac Regeneration Laboratory, Murdoch Children’s Research Institute, Flemington Road, Parkville, Melbourne, 3052 Australia; 2 Children’s Cancer Centre, The Royal Children’s Hospital, Flemington Road, Parkville, Melbourne, 3052 Australia; 3 Department of Pediatrics, University of Melbourne, Flemington Road, Parkville, Melbourne, 3052 Australia; 4 Cardiology Department, The Royal Children’s Hospital, Parkville, Melbourne, 3052 Australia; 5 Sydney Medical School, University of Sydney, Sydney, NSW 2050 Australia; 6 Dept of Cardiology, RPA Hospital, Sydney, NSW 2050 Australia; 7 Clinical Research Domain, Baker Heart and Diabetes Institute Commercial Road, Melbourne, VIC 3004 Australia; 8 Chris O’Brien Lifehouse Camperdown, Sydney, NSW 2050 Australia; 9 Kids Cancer Centre, Sydney Children’s Hospital Randwick, Sydney, Australia; 10 Discipline of Pediatrics and Child Health, School of Clinical Medicine, UNSW Medicine & Health, UNSW, Sydney, Australia; 11 Children’s Cancer Institute, Lowy Cancer Research Centre, UNSW, Sydney, Australia; 12 Division of Pediatric Hematology/Oncology, Children’s Mercy Kansas City, 2401 Gillham Road, Kansas City, MO 64108 USA; 13 Cancer Centre for Children, The Children’s Hospital, Corner of Hawkesbury Road and Hainsworth Street, Westmead, NSW 2145 Australia; 14 Department of Geriatrics, University of Melbourne, Parkville, Melbourne 3052 Australia; 15 Werribee Mercy Hospital, Werribee, Melbourne Australia; 16 The Heart Centre for Children, The Sydney Children’s Hospital Network Children’s Hospital, Westmead, Sydney, NSW Australia; 17 Medicine and Dentistry Clinical School, The University of Sydney, Sydney, NSW Australia

**Keywords:** Pediatric cardio-oncology, Pediatric cancer, Pediatric oncology, Cardio-oncology, Hypertension, Left ventricular dysfunction, Cascade testing

## Abstract

**Background:**

Modern oncological therapies together with chemotherapy and radiotherapy have broadened the agents that can cause cardiac sequelae, which can manifest for pediatric oncology patients while on active treatment. Recommendations for high-risk patients who should be monitored in a pediatric cardio-oncology clinic have previously been developed by expert Delphi consensus by our group. In 2022 we opened our first multidisciplinary pediatric cardio-oncology clinic adhering to these recommendations in surveillance and management.

**Objectives:**

Our pediatric cardio-oncology clinic aimed to:

(i) Document cardiovascular toxicities observed within a pediatric cardio-oncology clinic and.

(ii) Evaluate the applicability of the Australian and New Zealand Pediatric Cardio-Oncology recommendations.

**Methods:**

Monthly multidisciplinary cardio-oncology clinics were conducted in an Australian tertiary pediatric hospital. Structured standardised approaches to assessment were built into the electronic medical record (EMR). All patients underwent baseline echocardiogram and electrocardiogram assessment together with vital signs in conjunction with standard history and examination.

**Results:**

Nineteen (54%) individuals had a documented cardiovascular toxicity or pre-existing risk factor prior to referral. The two most common cardiovascular toxicities documented during clinic review included Left Ventricular Dysfunction (LVD) and hypertension. Of note 3 (8.1%) patients had CTCAE grade III LVD. An additional 10 (27%) patients reviewed in clinic had CTCAE grade I hypertension. None of these patients had hypertension noted within their referral. Cascade testing for cardiac history was warranted in 2 (5.4%) of patients.

**Conclusions:**

Pediatric cardio-oncology clinics are likely beneficial to documenting previously unrecognised cardiotoxicity and relevant cardiac family histories, whilst providing an opportunity to address lifestyle risk factors.

**Supplementary Information:**

The online version contains supplementary material available at 10.1186/s40959-023-00194-x.

## Introduction

Recent advances in pediatric oncology have seen overall survival rates increase to 85% in the developed world [1]. With more individuals surviving, therapeutically induced cardiovascular disease and its complications are an emerging problem for both oncologists and cardiologists. Studies show that childhood cancer survivors are at a 15-fold increased risk of developing congestive cardiac failure and have a 7-fold higher risk of premature death as compared to the general population [2]. In response, the field of cardio-oncology has emerged with increasing recognition that modern therapies have broadened the agents that can cause cardiac sequelae.

Our pediatric cardio-oncology clinic was established in May 2022 embedded within the outpatient oncology clinic of a pediatric tertiary institute that receives approximately 250 new pediatric cancer referrals per year. The multidisciplinary clinic comprises two pediatric oncologists, an academic pharmacist, a pediatric cardiologist and cardiology technologist.

The clinic was established to assess high-risk individuals as defined by the Australia and New Zealand Pediatric Cardio-Oncology Guidelines [3] using a structured approach to assessment, therapy modifications and surveillance.

This paper aims to

(i) Document cardiovascular toxicities observed within a pediatric cardio-oncology clinic and.
(ii) to evaluate the applicability of the Australian and New Zealand Pediatric Cardio-Oncology guidelines previously published by this group.

## Methods

### Individual cohort and referral approach

Individuals were referred to the clinic by pediatric oncologists, nurse co-ordinators or junior medical staff or identified through multi-disciplinary weekly team meetings across tumour streams. Referrals were based on the Australia and New Zealand Pediatric Cardio-Oncology referral guidelines (Table 1) [3]. Individuals could be referred for indications outside of these guidelines at the discretion of the referrer.

**Table 1:** Australian and New Zealand Pediatric Cardio-Oncology recommended indications for referral to a pediatric cardio-oncology clinic

Domain	Consensus definitions / Approach
**Domain 1**	*Defining high-risk pediatric oncology patients that should be reviewed by experts in cardio-oncology during acute therapy.* An individual will be considered high-risk if: ▪ they have received a total cumulative dose ≥250 mg/m^2^ (doxorubicin equivalent). ▪ the individual has relapsed and the cumulative doxorubicin equivalent dose (as part of first- or second-line therapy) will be ≥250 mg/m^2^. ▪ they have received any dose of anthracycline combined with radiotherapy ≥15Gy and where any area of the heart is involved in the treatment field as part of first- or second-line therapy. ▪ they have received radiotherapy ≥35Gy and where any area of the heart is involved in the treatment field as part of first- or second-line therapy. ▪ they have pre-existing congenital heart disease, a relevant family history of cardiovascular disease (including genetic disorders that impact heart structure and storage disorders but excluding adult-type cardiac disease i.e., myocardial ischemia, coronary artery disease etc) and those with previous abnormal left ventricular dysfunction. ▪ they are receiving treatment with VEGF inhibitors, mTOR inhibitors, proteasomal inhibitors, checkpoint inhibitors. They should ideally be seen at least once within a cardio-oncology clinic (if facilities exist), or more frequently to manage any potential associated cardiotoxicities as evidence emerges. ▪ in circumstances where the clinician screens for metabolic syndrome in pediatric cancer individuals and are diagnosed with metabolic syndrome. ▪ they have chronic kidney disease. ▪ they are an adolescent or young adult individual who is pregnant whilst receiving cancer therapy.

Recommendations for high-risk pediatric oncology patients who should be referred to a cardio-oncology clinic

### Cardio-oncology clinic structure

The cardio-oncology clinics were held monthly to a maximum of 4 new individuals and 3 returning individuals based within a dedicated outpatient oncology clinic in a tertiary pediatric hospital, as is typical to the public health care system in Australia.

### Clinic assessment and standardised templates

On arrival, individuals were seen by a cardiac technologist who performed a 2-dimensional echocardiogram and electrocardiogram (ECG). Echocardiography was performed as per standard guidelines from the American Society of Echocardiography [4]. The individual was then assessed by the pediatric oncologist and cardiologist (together) followed by the academic pharmacist. Aside from the echocardiogram and ECG, additional routine baseline assessment included anthropometric assessments, vital signs including manual blood pressure measurement using an age-appropriate blood pressure cuff a medical history, and physical examination. Following clinic review bloods were ordered for HbA1c and lipid profile, if not previously or recently performed as part of their metabolic screening.

An EMR SmartText was developed assist data capture and standardisation. Full SmartText annotations are shown in the Supplementary Data. The SmartText included (i) indication for referral; (ii) cumulative doxorubicin equivalent anthracycline dose; (iii) molecular therapy or CAR-T cells; (iv) family history of cardiac disease; (v) oncology treatment approach including irradiation doses; (vi) echocardiogram results; (vii) ECG result; (viii) cardiac MRI result; (ix) grading of cardiovascular toxicities; (x) metabolic syndrome screening; (xi) anthropometry measurements; (xii) medication reconciliation.

### Management and follow-up

Cardiovascular toxicities were graded according to the Common Terminology Criteria for Adverse Events (CTCAE) [5]. Management was determined according to published international guidelines for the given toxicity. Surveillance followed the recommendations from the Australia and New Zealand Pediatric Cardio-Oncology Guidelines [3]. Any follow-up tests were carried out within the tertiary institute at a time suitable to the individual and family.

Following each clinic, a multi-disciplinary meeting was held to discuss each individual and their management and surveillance recommendations. A summary letter was generated detailing the review and recommendations which were then distributed to the primary treating clinician.

### Research studies

Several research studies are offered within the cardio-oncology clinics. These research studies are part of the Australian Cardio-Oncology Biobank and Registry (ACOR) [6] and include studies into (i) pharmacogenomic predisposition; (ii) novel imaging; and (iii) digital health. Participation in the research studies is voluntary and independent of the clinic review.

### Data collection

Data was collected from the EMR or ACOR RedCap database. Reporting fields were standardised but designed with predictive branching to enable ease of reporting. The cardio-oncology clinic EMR SmartText was also built with this in mind to reduce time spent in data collection.

### Statistics

Data were collated and described with summary statistics. 95% confidence intervals were derived (where reported) using the Clopper-Pearson (exact) method.

## Results

### General information

There were thirty-seven referred individuals with a total of 62 clinic attendances. Of the 37 individuals seen 33 consented to their data being collected as part of the ACOR.

Twenty-two individuals (59.5%) were male and fifteen (40.5%) were female. Age at first clinic presentation and primary diagnosis are outlined in Table 2. Twenty-four (64.8%) individuals were receiving active oncology treatment at the time of referral (‘on treatment’). Thirteen (35%) individuals were ‘off treatment’. Nineteen (54%) individuals had a documented cardiovascular toxicity or pre-existing risk factor prior to referral including hypertension (1, 2.7%), Left Ventricular (LV) dysfunction (12, 32.4% of which one had a fascio-cutaneous skeletal syndrome with hypertrophic cardiomyopathy), new murmur (1, 2.7%), pericardial effusion (1, 2.7%), elevated troponin I (1, 2.7%) and tachycardia (1, 2.7%), genetic channelopathy (1, 2.7%), and (1, 2.7%) with x-linked intellectual disability syndrome (Alpha-thalassemia mental retardation (ATRX) syndrome) with a large atrial septal defect. A family history of cardiovascular disease was found, although this was not the reason for referral, in 9 (24.3%) individuals. Of note, one individual had a familial gene mutation causing a sodium channel dysfunction (SCN5A) known to be associated with cardiac conduction dysfunction, dilated cardiomyopathy, Brugada syndrome and prolonged QT syndrome [7]. Another family had a history of a bicuspid aortic valve (father of referred individual) not previously recorded in the EMR. This triggered cascade testing in the family, whereby the first-degree relatives of the principal patient were screened for a bicuspid aortic valve (Table 3).

**Table 2:** Demographics, referral indications and cardiovascular disease identified during cardio-oncology clinic

	N=	Percent
Sex		
Male	22	59.5%
Female	15	40.5%
Age	37	
0–5	3	8.1%
5–10	10	27%
10–15	13	35.1%
15–18	11	29.7%
Disease	37	
Leukaemia Post HSCT	9	24.3%
Ph + ALL Post HSCT	2	5.4%
ALL	6	16.2%
AML	4	10.8%
Hodgkin’s Lymphoma	3	8.1%
Non-Malignant Disease Post HSCT	2	5.4%
Sarcoma	4	10.8%
Langerhans Cell Histiocytosis	1	2.7%
PVL	1	2.7%
Brain tumour	2	5.4%
Wilm’s Tumour	1	2.7%
APML	2	5.4%
Reason for Referral		
Family history of genetic cardiac disease	1	2.7%
Hypertension	1	2.7%
New Murmur	1	2.7%
CAD > 250 mg/m^2^ and TKI	2	5.4%
CAD > 250 mg/m^2^ and immunotherapy	1	2.7%
CAD > 250 mg/m^2^ (off treatment)	5	13.5%
AYA pregnant after cancer therapy	1	2.7%
Tyrosine Kinase Inhibitors	1	2.7%
BRAF inhibitor	2	5.8%
Pre-existing or elevated biomarkers	1	2.7%
Left ventricular dysfunction (on treatment)	9	24.3%
Left ventricular dysfunction (off treatment prior TKI)	1	8.1%
Left ventricular dysfunction (off treatment)	2	5.8%
Any dose anthracycline and radiotherapy >15Gy with mediastinum in field	1	2.7%
Risk of QTc prolongation	1	2.7%
Other cardiac indication	2	5.4%
Off treatment (other indication)	3	8.1%
Family HX Bicuspid Valve	2	5.4%
Cardiovascular disease identified in clinic		
Hypertension	12	32.4%
Myocardial Infarction	0	0%
QTc prolongation	2	5.4%
Atrial Fibrillation	0	0%
Accelerated atherosclerosis	0	0%
Conduction disorder	0	0%
Arterial thrombotic event	0	0%
Left ventricular dysfunction	7	18.9%
Aortic Regurgitation	1	2.7%
Metabolic Syndrome	1	2.7%
BVD Needing Cascade Testing	2	5.4%
Other	2	5.4%

Abbreviations: *ALL* acute lymphoblastic leukaemia, *AML* acute myeloid leukaemia, *APML* acute promyelocytic leukaemia, *AYA* Adolescent and young adult, *BMF* Bone Marrow Failure, *BVD* Bicuspid Valve Disorder, *CAD* Cumulative Anthracycline Dose, *HSCT* Haematopoietic stem cell transplant, *MTOR* mammalian target of rapamycin, *PVL* pigmented villonodular synovitis, *SCD* sickle cell disease, *TKI* tyrosine kinase inhibitor, *VEGF* vascular endothelial growth factor

**Table 3:** Cardiovascular toxicities documented during cardio oncology clinic assessment

CTCAE Grade		(n)	%
	**Hypertension**	**12**	**34**
0	Not present	23	66
1	Systolic/diastolic blood pressure > 90th percentile but <95th percentile	10	29
2	Systolic/diastolic blood pressure > 95th percentile but <99th centile	2	6
3	Systolic/diastolic blood pressure > 5mmhg above 99th percentile	0	0
4	Life threatening consequences (i.e., malignant hypertension)	0	0
	**QTc Prolongation**	**2**	**6**
0	Not present	33	94
1	Average QTc 450 – 480 ms	2	6
2	Average QTc 481 – 500 ms	0	0
3	Average QTc ≥ 501 ms; > 60 ms change from baseline	0	0
4	Torsades de pointes; polymorphic ventricular tachycardia etc	0	0
	**Left Ventricular Systolic Dysfunction or Ejection Fraction Decreased**	**9**	**24.3**
0	Not present	27	77
1	Ejection Fraction Decreased < 50–40% or < 10–19% drop from baseline	4	11
2	Ejection Fraction Decreased 50–40% or 10–19% drop from baseline	1	3
3	Symptomatic due to drop in ejection fraction responsive to intervention	3	9
4	Refractory or poorly controlled heart failure due to drop in ejection fraction; intervention such as ventricular assist device, vasopressor support or heart transplant indicated	0	0
	**Atrioventricular Block First degree**	**1**	**2.7**
0	Not present	36	97.3
1	Asymptomatic, intervention not indicated	1	3
2	Non-urgent intervention indicated	0	0
	**Aortic Valve Disease**	**1**	**2.7**
0	Not present	36	0
1	Asymptomatic valvular thickening with or without mild valvular regurgitation or stenosis by imaging	1	3
2	Asymptomatic; moderate regurgitation or stenosis by imaging	0	0
3	Symptomatic; severe regurgitation or stenosis by imaging; symptoms controlled with medical intervention	0	0
4	Life-threatening consequences; urgent intervention indicated (i.e., valve replacement, valvuloplasty)	0	0

### Indication for referral to clinic

The most common reasons for referral to the clinic was previous exposure to a cumulative doxorubicin equivalent anthracycline dose ≥250 mg/m^2^ (*n* = 9 (24.3%) with 6 individuals off treatment, 3 on treatment) and left ventricular dysfunction (LVD) often in individuals who had a background exposure of ≥250 mg/m^2^ anthracycline (*n* = 12 (32.4%), 9 on treatment, 3 off treatment). The median cumulative doxorubicin equivalent anthracycline dose exposure of 150 mg/m^2^(interquartile range 26 – 480 mg/m^2^). Five of these individuals had evidence of prior cardiac dysfunction that had recovered on serial 2-dimensional echocardiography imaging.

Molecular inhibitors (monotherapy or in combination with chemotherapy) were another common reason for referral (*n* = 6, 16.2%) including tyrosine kinase inhibitor use (in combination with other referral indications) (*n* = 4, 10.8%) or BRAF inhibitor use (*n* = 2, 5.4%). Seven (18.9%) individuals had a history of radiation although it was only listed as primary reason for referral in 1 individual (2.7%). Less frequent referral indications included family history of genetic heart disease (*n* = 1, 2.7%) and genetic syndrome with congenital heart disease diagnosis (n = 1, 2.7%).

Several referrals were made for indications outside of guidelines [3]. Individuals were referred for (i) previously elevated biomarkers (n = 1, 2.7%); (ii) tachycardia (n = 1, 2.7%); pericardial effusion (n = 1, 2.7%); (iii) hypertension without metabolic syndrome (n = 1, 2.7%); (iv) newly identified murmur (n = 1, 1.7%); (vi) risk of QTc prolongation as a result of arsenic trioxide exposure (*n* = 2, 5.4%).

### CTCAE graded cardiovascular toxicities documented during clinic attendance

**Fig. 1:**
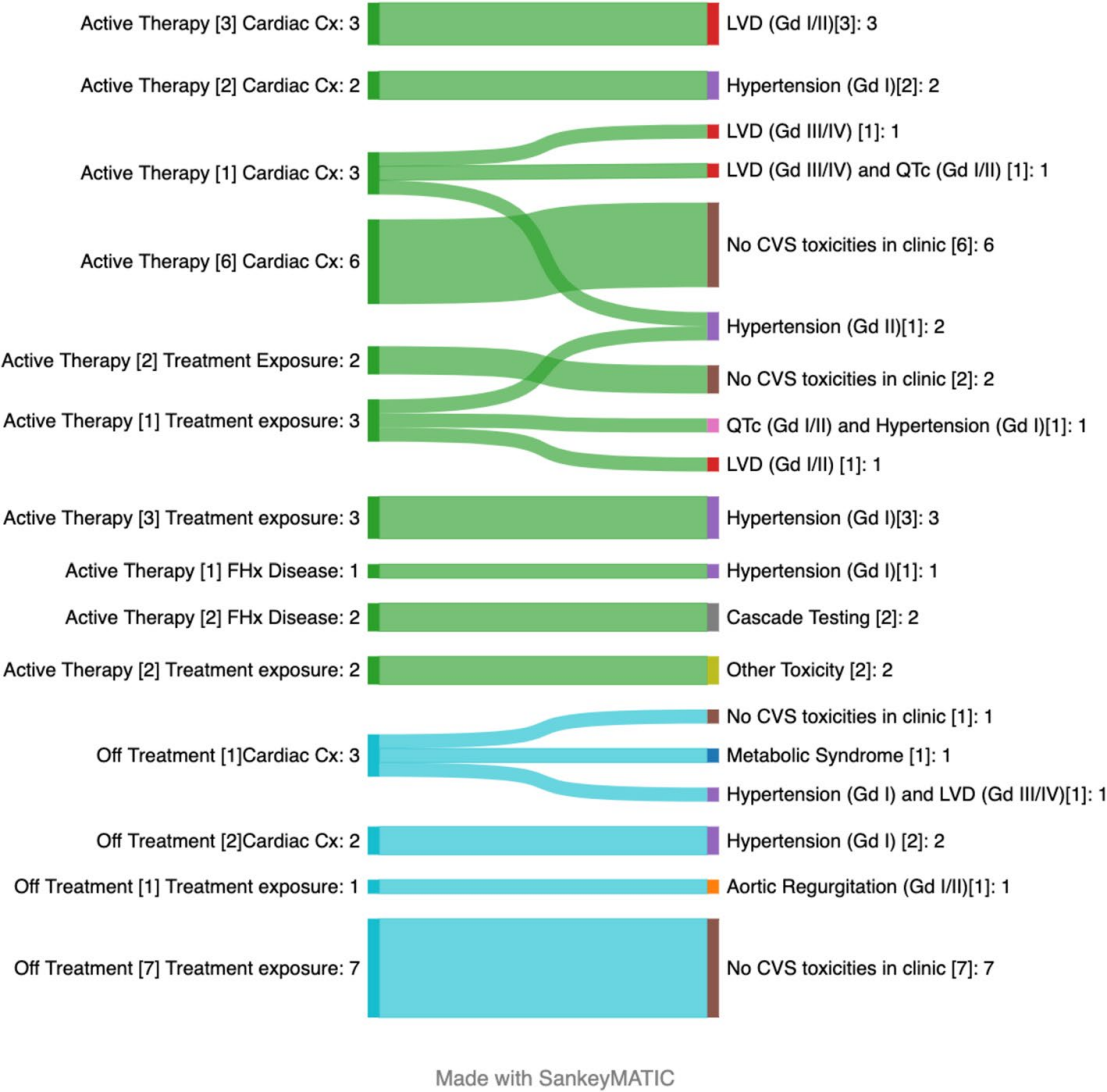
Referral indications and graded cardiovascular toxicities observed at the time of clinic attendance. Referral indications for patients during “active therapy” and those “post therapy” subgrouped according to their referral indication: [1] Treatment exposure (anthracycline dose, radiotherapy, targeted therapy included in domain 1 of guidelines) versus [2] Cardiac complications (LVD, troponin elevation, hypertension, QTc prolonged, murmur) versus History (FHx, or personal Hx). Abbreviations: Cx; Complication: FHx; Family history LVD: Left Ventricular Dysfunction; QTc: QTc prolonged]

The referral indication and graded cardiovascular toxicities are shown in Fig. 1.

### Left ventricle (LV) dysfunction (LVD)

Twelve (32.4%) individuals were referred for a prior history of LVD demonstrated on echocardiography, nine (75%) of which occurred during active therapy and three occurred off therapy (25%). Of the twelve individuals with LVD, three (25%) had CTCAE grade III-IV dysfunction while three individuals (25%) had a previous drop in ejection fraction (EF) (CTCAE grade I-II) that had resolved at the time of clinic appointment.

One individual with grade III-IV LVD had received a cumulative doxorubicin equivalent anthracycline dose of 237 mg/m^2^ without dexrazoxane. On referral, they had a fractional shortening (FS) of 22.4% and EF (Teichholz) 45.5% (Simpson biplane LV EF unable to be performed due to technical issues). The individual had been referred at the end of therapy and cardiac function measurements during cardio-oncology clinic confirmed CTCAE grade I heart failure and CTCAE grade III for LVD. An Angiotensin Converting Enzyme Inhibitor (ACEI) was commenced, and the individual remains on surveillance.

A second individual was referred with a history of LVD (CTCAE grade III) initially found while on active treatment, that required commencement of an ACEI for heart failure (CTCAE grade I). The individual’s echocardiogram prior to commencement of an ACEI demonstrated a FS of 22.9%, EF(Teichholz) 46.1% and a Simpson biplane LV EF 47%. The individual had a history of Acute Myeloid Leukaemia (AML) with a secondary myelodysplastic syndrome requiring an allogeneic transplant. The individual had a cumulative doxorubicin equivalent anthracycline dose of 840 mg/m^2^, receiving dexrazoxane during therapy for their secondary cancer only. Magnetic resonance imaging confirmed abnormal LV function and abnormal epicardial and mid-wall myocardial fibrosis of the basal LV wall affecting 5% of the total LV myocardium. This individual remains clinically stable on an ACEI with ongoing surveillance.

One individual had been referred with known grade III LVD on a background of Noonan-Costello syndrome associated with hypertrophic cardiomyopathy. The individual was being treated with imatinib on referral and remained stable on surveillance.

Of the remaining individuals, three (9%) demonstrated a CTCAE grade II decrease in EF resulting in the multi-disciplinary team recommending the commencement of dexrazoxane with all future doses of anthracycline and early follow-up examination. One individual who was post stem cell transplant on sorafenib had a history of grade II decrease in EF however was discharged from clinic back to their primary oncologist for ongoing routine surveillance once they completed their sorafenib therapy. A further two (6%) individuals were identified as not having had a true decline in EF or indeed LVD on review of their initial echocardiogram. The first was referred with an EF 46% and due to their body habitus limiting echocardiographic views, subsequently underwent a cardiac MRI to confirm their cardiac function (body mass index 32, weight 99th centile). The cardiac MRI demonstrated normal cardiac function with no signs of myocardial fibrosis or oedema and the individual was discharged back to their primary oncologist. The second individual was referred for concerns regarding early diastolic dysfunction on initial echocardiogram imaging prior to anthracycline exposure (EF 48%) however, review of the individual’s previous echocardiograms as well as repeated echocardiogram imaging in clinic, did not confirm initial findings and the individual was discharged back to their oncologist for routine surveillance.

### Hypertension

Hypertension was screened for in all individuals as part of the clinic assessment. Twelve patients were found to have an elevated blood pressure has per CTCAE guidelines. Of these 12, 5 (41.6%) patients were on treatment. Of note, 9 patients (75%) diagnosed with either grade 1 or grade 2 hypertension had a leukemia diagnosis.

Screening assessments found ten (27.0%) individuals had blood pressures between the 90th and 95th percentile for age, sex and height (CTCAE grade I). Hypertension was presumed to be secondary to molecular inhibitor use in 3 (8.1%) individuals. Of note, whilst blood pressures were recorded automatically in the medical notes, further screening recommendations for CTCAE grade I hypertension were not documented in the EMR.

Two further individuals (5.4%) were found to have blood pressures between the 95th and 99th percentile (CTCAE grade II) within clinic, not previously noted in the referral or on EMR. Only 1 of the 2 individuals demonstrated persistent grade 2 hypertension. The individual with grade 2 hypertension had a background diagnosis of Acute Lymphoblastic Leukaemia (ALL). At the time of hypertension documentation, the individual was receiving corticosteroids. The hypertension was successfully managed with a calcium channel blocker. Their cardiac function and ECG assessments performed during clinic were within normal limits and hypertension was the individuals only identified cardiovascular toxicity. The second individual was referred for hypertension and already on an anti-hypertensive agent with normal blood pressure documented in the cardio-oncology clinic.

### Prolonged QTc measurement or arrhythmias

Evaluation for QTc prolongation was performed in all individuals. QTc prolongation was only demonstrated in two (5.4%) individuals and was documented as CTCAE grade I (both measured at a median of 470 ms). One individual was on maintenance tyrosine kinase inhibitor therapy post bone marrow transplant with stable QTc. The second was an individual diagnosed with medulloblastoma. The underlying cause of the individuals prolonged QTc was uncertain with no medication precipitants identified. However, the individual also had a background diagnosis of Fanconi syndrome that was thought to be contributory.

The referring teams for individuals at risk of QTc prolongation were provided with individual specific multidisciplinary written information on how to best minimise the risk of prolonged QTc and surveillance guidance.

### Metabolic disease screening

One individual met criteria for metabolic syndrome. The individual had completed treatment for T-cell ALL and was referred to clinic for LVD. The individual had a long history of morbid obesity and many multiple failed attempts to reduce weight. The individual was counselled on modifiable lifestyle factors and was referred to our institute’s obesity clinic for management. Although only one individual met criteria for metabolic syndrome, all individuals were counselled on the importance of modifiable lifestyle factors with respect to cardiovascular disease.

### Interventions undertaken in cardio-oncology clinic

**Fig. 2:**
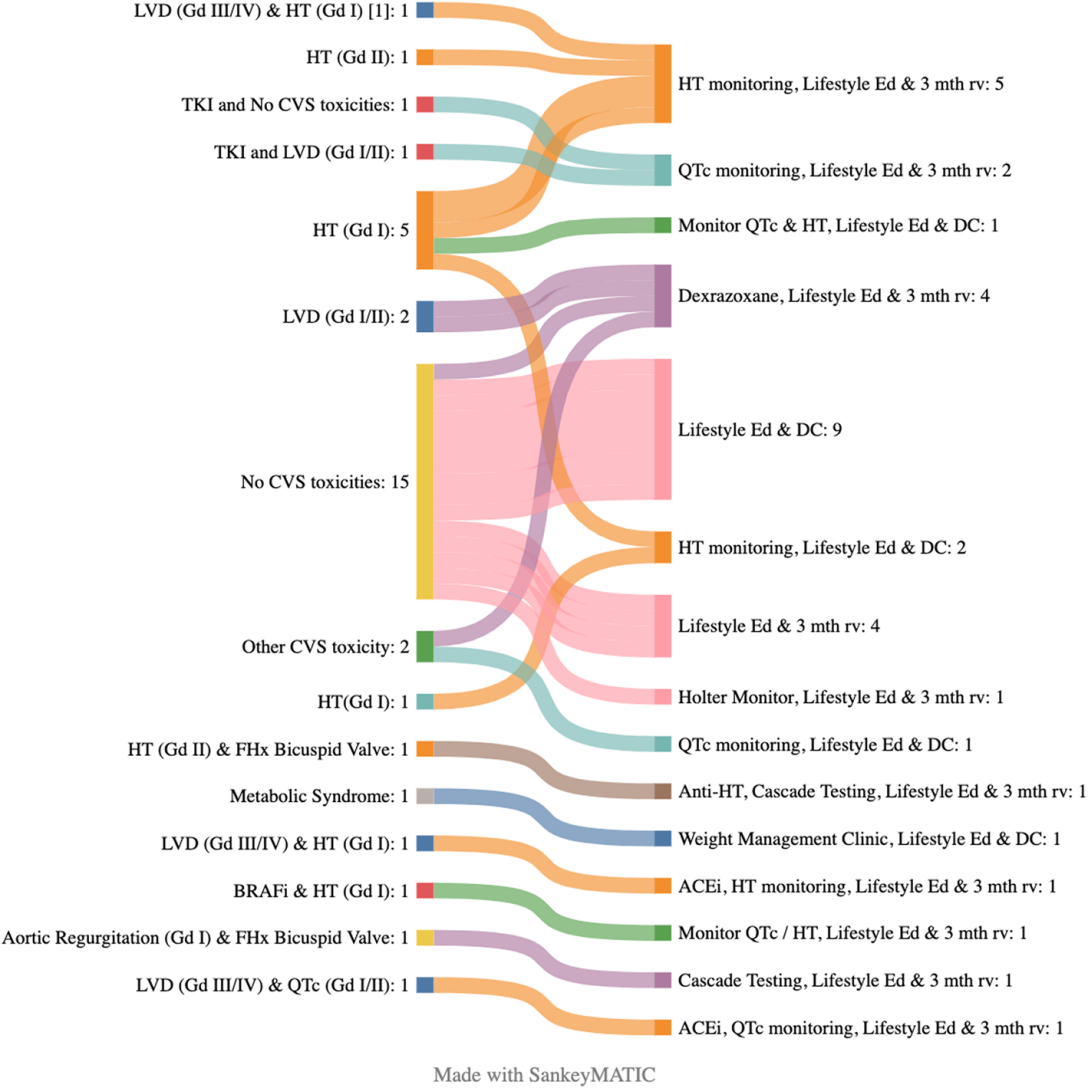
Cardiovascular toxicities documented in clinic and subsequent interventions and surveillance recommendations. Sankey graph showing graded cardiovascular toxicities within the pediatric cardio-oncology clinic and subsequent recommendations and interventions. Abbreviations: ACEi: Angiotensin converting enzyme inhibitor; BRAFi: BRAF inhibitor; DC; Discharge from clinic; FHx; Family history; HT: Hypertension; Lifestyle Ed: Lifestyle Education; LVD: Left Ventricular Dysfunction; QTc: QTc prolonged; TKI: Tyrosine kinase inhibitor, 3 mth rv: 3 month review

Of the 37 individuals referred to clinic during the year, 23 (62.1%) remain on ongoing surveillance in the cardio-oncology clinic (Fig. 2). The interventions undertaken in clinic were many and varied. For the individuals remaining in surveillance in the cardio-oncology clinic (*n* = 23) interventions and advice included addition of dexrazoxane (*n* = 4, 17.4%), Holter monitoring (*n* = 1, 4.3%), hypertension monitoring (*n* = 6, 28.5%), cascade testing for familial valvular disease (n = 2, 8.6%), QTc monitoring (n = 6, 26%) and lifestyle advice (n = 23, 100%).

For the individuals who were discharged from clinic (*n* = 14) the interventions and advice included ongoing QTc monitoring (n = 1, 7.1%), hypertension monitoring (*n* = 3, 21.4%), referral to weight management clinic (n = 1, 7.1%), and further lifestyle advice on modifiable cardiovascular risk factors (n = 14, 100%). Overall, 27 individuals (72.9%) seen within this multidisciplinary clinic had interventions or monitoring recommendations not previously made in their routine oncology care.

### Referral indications grouped according to whether the individual met criteria for referral based on the current Australian and New Zealand pediatric cardio-oncology guidelines

**Fig. 3:**
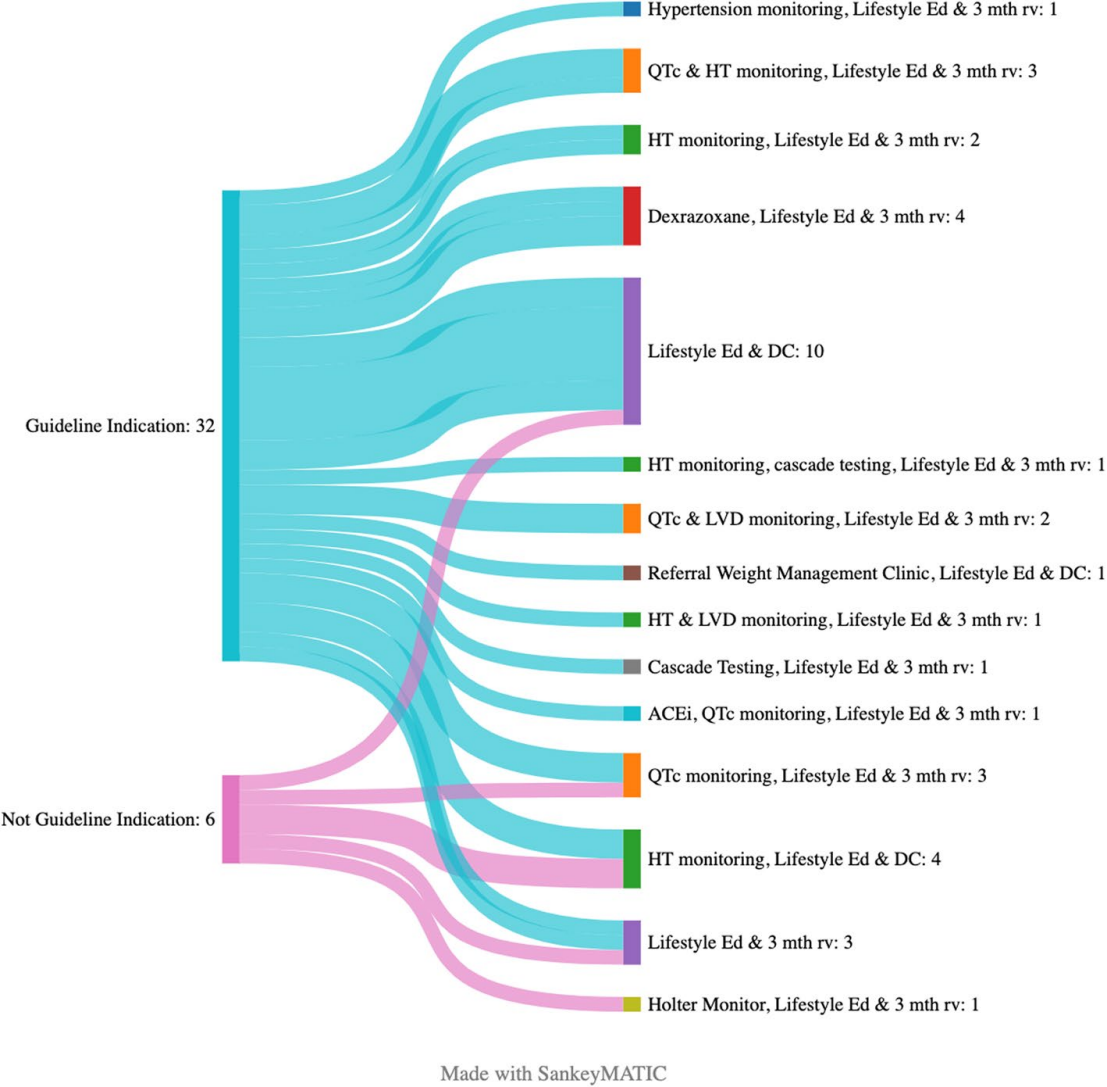
Referral guideline concordance and interventions/monitoring recommendations. Referral indications categorised according to whether the referral indication is in concordance with the Australian and New Zealand Pediatric Cardio Oncology recommendations for cardio-oncology clinic referral. Interventions and monitoring recommendations made in cardio-oncology clinic are listed according to referral indication. Abbreviations: ACEi: angiotensin converting enzyme inhibitor; DC: Discharge; Ed: Education;3 mth rv: three month review; HT: Hypertension

Of the individuals referred to clinic, 31 met the cardio-oncology clinic referral criteria according to the current Australian and New Zealand Pediatric Cardio-Oncology guidelines [3] (Fig. 3). An additional six individuals were referred for exhibiting symptoms or signs that potentially could indicate a cardiovascular toxicity but are not included in the current guidelines in isolation and included palpitations, tachycardia, prolonged QTc in the absence of being on a molecular targeted agent. Of these individuals, 3 (50%) continue to need ongoing review in the cardio-oncology clinic with interventions including hypertension monitoring (*n* = 1), QTc monitoring (n = 1) and Holter monitoring (n = 1). Therefore of 37 referrals to the clinic, 3 were outside the referral guideline but benefited from review in the clinic for a proportion of 0.081 with a 95% confidence (interval of 0.017 to 0.22).

### Applicability of the Australian-New Zealand pediatric cardio-oncology guidelines

Review of practice during first year of clinic demonstrates that Domain 2 of the guidelines were generally applicable. Baseline measurements with history, examination and point of care testing were adhered to for all patients. The same imaging modality (standard 2D echocardiography) were used for all patients and access to cardiac MRI was available when deemed clinically necessary. During the time of this data collection, only one patient was able to be assessed with global longitudinal strain due to limitations within the hospital. All other domains were followed as per the guidelines, where applicable, for those reviewed in the clinic.

## Discussion

Despite improvements in the overall survival of children with cancer, therapy related cardiovascular toxicity remains a significant cause of morbidity [8, 9]. Here, we present data from the first pediatric cardio-oncology service to be established in the Australia-Pacific region, with at-risk group, monitoring and surveillance recommendations based on the Delphi consensus guidelines [3].

The two main cardiovascular toxicities observed in clinic included LVD and hypertension. Among the individuals reviewed following referral for LVD, 5 of the 12 individuals had resolution of their reduced EF on echocardiogram. Interestingly, in a recent study of 787 individuals of young adult populations treated with trastuzumab in the adjuvant setting of HER2-positive breast cancer, a drop of > 5% LV EF within the first 3 months of treatment, even with subsequent normalisation of function, was strongly associated with later development of trastuzumab related cardiotoxicity [10]. Similarly, Nousiainen et al., showed in adult individuals with non-Hodgkin’s Lymphoma, an early decline in LV EF (< 50%) predicts the later decrease in LV EF and cardiomyopathy [11]. In pediatric oncology cohorts, Lipshultz et al. demonstrated that past anthracycline-associated cardiotoxicity often predicts future cardiomyopathy development [12]. As such, individuals seen within our clinic who had previously documented LVD, continue to be seen at least every 6 months to monitor for a second decline in function. Furthermore, 2 individuals commenced on ACEI in the setting of grade III LVD, with a further individual already receiving ACEI. This approach is to prevent pathological LV remodelling using medications that target either preload (diuretics) or afterload (ACE inhibitors or angiotensin-receptor blockers) [12]. It is well recognised that the therapeutic benefit of these medications is transient and when to cease their use remains controversial.

The known association between molecular inhibitors and cardiovascular side effects was observed in clinic. Indeed, hypertension was the second most common cardiovascular toxicity observed in clinic. Grade I hypertension was seen in 10 (27%) of individuals attending clinic of which 3 (33%) were taking a molecular inhibitor. The exact molecular mechanisms underlying hypertension with respect to molecular inhibitors remain unclear, although links have been made to oxidative stress, endothelial dysfunction, increased sympathetic outflow, and reduced nitric oxide generation [13]. To date, insufficient evidence exists to support a hypertension guideline specific to anti-cancer therapies and therefore the therapeutic approach we followed was in line with the American Academy of Pediatrics (AAP) [13]. It is important to note that this staging system does not precisely concur with the CTCAE grading.

### Family history

This clinic has identified the utility of a dedicated time to review cardiovascular family history. Surprisingly, of the 26% of individuals who had a history of cardiac disease, 88% did not have this information recorded in the EMR. Concerningly, 3 of these individuals had cardiac histories that warranted intervention, closer follow-up in childhood or cascade testing of their family members. A recent publication by the St Jude’s Lifetime Cohort report found that a cardiovascular family history increases the risk of late-onset cardiovascular outcomes in childhood cancer survivors [14]. In this study, (*n* = 1260) having a first degree relative with atherosclerotic disease was independently associated with the development of treatment related heart failure. Having a first degree relative who had hypertension or any cardiovascular disease (i.e. myocardial infarction, stroke or heart failure) was associated with an increased risk of developing hypertension in childhood cancer survivors. Therefore, an accurate family history can assist in assessing an individual’s risk of long-term cardiovascular disease and managing modifiable risk factors.

Some cardiovascular histories can lead to cascade testing (as was seen in our clinic with family histories of bicuspid valves). According to the American Heart Association, individuals who have a bicuspid aortic valve should have all first-degree relatives (parents and siblings) screened for the disease. This is because bicuspid aortic valve can be managed prior to causing symptoms or complications [15]. Pediatric cardio-oncology is a relatively new specialty and whilst multiple papers have been written about the need for multi-disciplinary approaches, risk stratification, screening for congenital heart disease, and management of long-term lifestyle factors, guidance around the enquiry of and management of familial cardiovascular disease is largely omitted and an area for further research [16].

### Modifiable risk factors

Long-term modifiable risk factors were discussed with all individuals who attended the clinics. Under-diagnosis and under-treatment of modifiable cardiovascular risk factors in childhood cancer survivors has been recognised as a contributing factor to the increased cardiovascular morbidity in this high-risk group [17]. Physical inactivity contributes significantly to cardiovascular disease and metabolic syndrome [18]. Pediatric and adolescent young adult individuals with cancer are less physical active [19] than their aged-matched controls which is associated decreased quality of life [20]. One of the unforeseen benefits of the clinic was creating a dedicated time to specifically address modifiable cardiovascular risk factors outside of the oncology outpatient setting which is focussed upon acute and active treatment needs. Furthermore, in late effects clinics, whilst modifiable risk factors are addressed, these appointments are infrequent and many years off-treatment. Addressing modifiable risk factors earlier in a designated clinic provides an unprecedented opportunity to impact long-term behavioural change.

### Benefits of a multi-disciplinary clinic to the individual and to the practitioner

**Fig. 4:**
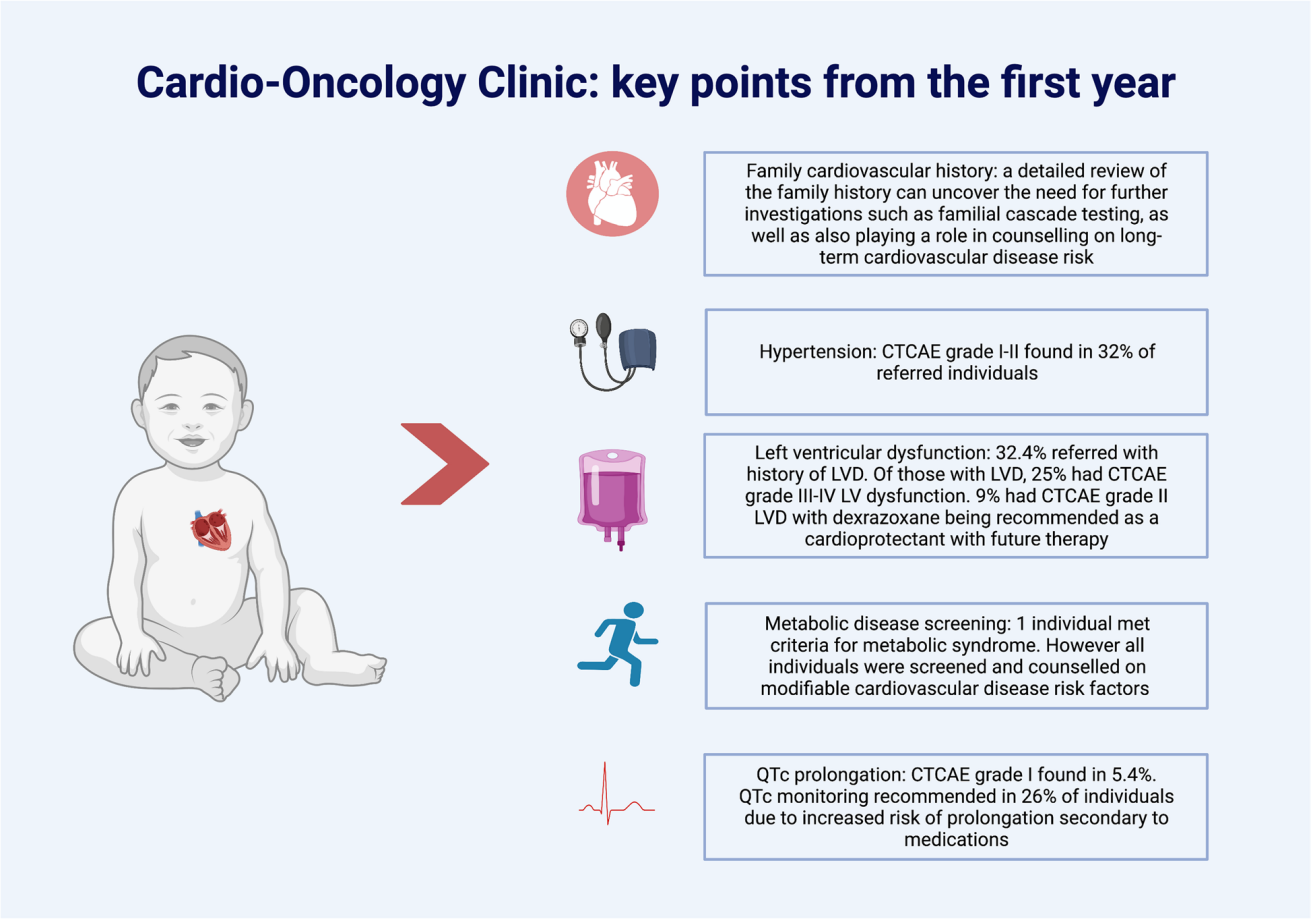
Cardio-Oncology Clinic: Key points from the first year. The most common observed cardiovascular toxicities were LVD (with 32.4% referred with a history of LVD and of those 25% had CTCAE grade III-IV LVD) and hypertension (where 32% of referred individuals were found to have CTCAE grade I-II hypertension). In addition, QTc prolongation was found in 5.4% of individuals and QTc monitoring was recommended in 26% of individuals. A detailed review of family history can uncover the need for cascade testing and targeted counselling

A dedicated cardio-oncology clinic provides the opportunity for the holistic cardiovascular management of an individual on active therapy. Our clinic works to assess and address each individual’s cardiovascular needs across their therapeutic journey (Fig. 4). The cross pollination between the multidisciplinary team members allows for sharing of expertise and education, both with the individuals and their families as well as health care professionals. Cardiovascular toxicity associated with cancer therapies is well documented [21–23], the establishment of a pediatric cardio-oncology clinic attempts to decrease the long-term burden of cardiac morbidities that are found in childhood cancer survivors by the early detection of cardiac dysfunction, advocating for the use of cardiac protectants on therapies (where feasible) and targeting modifiable lifestyle risk factors.

### Future directions

There are several areas for improvement in our second year of operation. Until recently, the reporting of global longitudinal strain (GLS) on echocardiogram was not available at our institute. GLS has previously been proposed as a more sensitive tool for LV EF, and there is favour for using this more reproducible approach along with 3D-LV EF and cardiac magnetic resonance [24]. In an era where detecting LVD earlier has potential therapeutic benefit, there is an urgency to drive the change to include GLS in standard reporting in pediatric oncology individuals both during and following treatment. Lead by the cardiologist within our clinic, GLS will now be routinely reported.

A second area for future development is the revision of the Australian and New Zealand Pediatric Cardio-Oncology recommendations developed via expert Delphi consensus in 2022 [3]. The development of the guidelines was for the purpose of cardiovascular monitoring during therapy, and not meant to replace surveillance or late effects guidelines for example, the Children’s Oncology Group Late Effects Guidelines or those from the International Guideline Harmonisation Group [25]. The first year of data highlights a need to revise some aspects of the guidelines particularly with respect to individuals who have developed signs or symptoms that may indicate a cardiac toxicity but do not meet any of the other current indications for referral. In our data, 3 individuals were referred for indications not currently in the guidelines but benefited from attendance, however there is substantial uncertainty about the true proportion. An indication can be gained from the 95% confidence interval for this proportion which lies from 1.7 to 22%. Further data collection from ongoing clinics in its second year will define a more precise estimate of the proportion of individuals outside guidelines who might benefit further from having attended the clinic.

Thirdly, the Australian and New Zealand pediatric cardio-oncology guidelines do not make any recommendations on the use of dexrazoxane. Recent published guidelines from the International Late Effects of Childhood Cancer Guideline Harmonisation Group summarise the evidence for the use of pre-emptive dexrazoxane [26]. No comment is made on the use of dexrazoxane in the setting of LV dysfunction. Updating the Australian and New Zealand pediatric cardio-oncology guidelines to reflect the current published evidence of dexrazoxane use and its use in the setting of dysfunction, will help to further standardise care.

Furthermore, while the intention of the clinic was to specifically monitor at risk patients during active treatment, the operation of the clinic saw that 35% were referred in their post-therapy period. Although this falls outside the scope of the Australian and New Zealand pediatric cardio-oncology guidelines, a subset of these patients proved to be of high risk of dysfunction and close monitoring within a specialised setting is logical. More follow up data is required to demonstrate whether this equates to early detection of dysfunction and improvement in outcomes.

### Limitations

Our study is limited by sample size secondary to first year of operation of the cardio oncology clinic. Thus, the findings need to be interpreted in this context and will benefit from further years of operation. Furthermore, although all attempts to ensure recruitment into the clinic were made by team members attending weekly stream specific multi-disciplinary meetings to ensure full capture of patients, there remains a risk that not all patients that were eligible for the clinic were referred. This limits the ability to fully assess the applicability of the Australian and New Zealand pediatric cardio-oncology guidelines. It is expected that as the profile of the clinic is raised, this potential gap in captured patients disappears.

## Conclusion

The pediatric cardio-oncology clinic provides a dedicated clinic to assess the cardiovascular needs of patients during cancer therapy. It provides the ability for the early detection of cardiac toxicity, the commencement of therapeutic interventions as well as an opportunity for education for health care professionals, parents and patients. The incorporation of more advanced imaging techniques, advocating for the use of harm minimisation oncological strategies and consumer education will work towards reducing the long-term cardiovascular risk of childhood cancer survivors.

### Supplementary Information

**Additional file 1.** Supplementary data.

## Data Availability

Access to deidentified data can be made upon individual request to the authors and upon approval by administering HREC.

## Abbreviations

ACEI: Angiotensin Converting Enzyme Inhibitor
AML: Acute Myeloid Leukaemia
CTCAE: Common Terminology Criteria Adverse Events
ECG: Electrocardiogram
EF: Ejection Fraction
EMR: Electronic Medical Record
FS: Fractional Shortening
LVD: Left Ventricular Dysfunction
MRI: Magnetic Resonance Imaging
