# Supplementary material for: Lessons learnt in the first year of an Australian pediatric cardio oncology clinic
Source: Cardiooncology. 2023 Dec 7;9:45. doi: 10.1186/s40959-023-00194-x (PMC10702043; doi:10.1186/s40959-023-00194-x)
Supplement: Supplementary file 1 — Additional file 1. Supplementary data. [file 40959_2023_194_MOESM1_ESM.docx]

**SUPPLEMENTARY DATA**

| **Main Heading** |  |  |
| --- | --- | --- |
| Reason for referral | - Cumulative dose anthracycline > 250mg/m^2^ doxorubicin equivalent or greater. - Radiotherapy ≥ 30Gy and including the heart. - Mediastinal radiation and any dose of anthracycline - Anthracycline. - LV dysfunction - VEGFi - MTORi - Proteasomal inhibitor - Checkpoint inhibitor - Pre-exicinting or elevated cardiac biomarkers - Prior exposure to cardiotoxic treatment - Co-morbities (i.e., diabetes, obesity, renal dysfunction, pulmonary disease, endocrinopathies, elctrolyte and metabolic abnormalities, pregnancy, virus) - *** (other) |  |
| Risk Factors and preclinical work-up | - HbA1c - Lipids - GFR - Past history CVS disease - Smoker - *** (other) |  |
| Diagnosis^##^ |  |  |
| Cancer Stage^##^ |  |  |
| Treatment Stage^##^ |  |  |
| Cumulative doxorubicin equivalent dose^##^ |  |  |
| Molecular inhibitor | - Yes - No - N/A - Date started *** |  |
| Checkpoint inhibitor | - Yes - No - N/A   Date started *** |  |
| Car-T cell recipient | - Yes - No - N/A   Date started *** |  |
| ECHOCARDIOGRAM/ECG^#^ |  |  |
| Echocardiogram result | - *** |  |
| ECG result | - *** |  |
| Cardiac MRI result | - *** |  |
| Indication cardiac MRI | - Yes - No - N/A |  |
| Performance score | - Lanksy score - Karnofsky score - ECOG performance status |  |
| Current medications^##^ |  |  |
| Cardiac examination |  |  |
| General appearance | - Healthy, alert, active, cooperative, and in no distress - Mild distress - Moderate distress - Severe distress - Uncooperative - Pale - Flushed - Crying - Inattentive - Listless - Not interactive with examiner - *** (other) |  |
| Recent laboratory findings^##^ | - Sodium - Potassium - Chloride - Bicarbonate - Urea - Creatinine - eGFR - Calcium - Magnesium - Phosphate - Lipase - Triglyceride - Glucose |  |
| Screened cardiovascular risk factors | - *** |  |
| Cardiac disease identified in clinic | - Hypertension - Myocardial infarction - QT prolongation - Atrial fibrillation - Accelerated atherosclerosis - Conduction disorder. - Arterial thrombotic events - Left ventricular dysfunction |  |
| Medication changes by academic pharmacist | - No change - Altered dose - Cease medications - Cardiovascular therapy recommended - *** (other) |  |
| Action/surveillance/Plan | - *** |  |
| Next review by cardio-oncology | - 1 month - 3 months - 6 months - 12 months - Discharge - *** (other) |  |

**Supplementary Data Table 1 :** **EMR Smart Text approach used in Clinic:** SmartText used during the first 12 months of clinical documentation within the institutions EMR. Abbreviations; EMR: Electronic Medical Record

^#^ Hyperlink to EMR results, ^##^ Automatically filled from EMR
